# Transcriptome and Proteome Association Analysis to Screen Candidate Genes Related to Salt Tolerance in *Reaumuria soongorica* Leaves under Salt Stress

**DOI:** 10.3390/plants12203542

**Published:** 2023-10-12

**Authors:** Hanghang Liu, Peifang Chong, Shipeng Yan, Zehua Liu, Xinguang Bao, Bingbing Tan

**Affiliations:** 1 College of Forestry, Gansu Agricultural University, Lanzhou 730070, China; liuhanghang131456@163.com (H.L.); liuzehua@gsau.edu.cn (Z.L.); baoxg131456@163.com (X.B.); tanbb131456@163.com (B.T.); 2 School of Forestry Engineering, Shandong Agriculture and Engineering University, Jinan 250100, China; 15379380889@163.com

**Keywords:** *Reaumuria soongorica*, salt stress, transcriptome, proteome, association analysis

## Abstract

This work aims at studying the molecular mechanisms underlying the response of *Reaumuria soongorica* to salt stress. We used RNA sequencing (RNA-Seq) and Tandem Mass Tag (TMT) techniques to identify differentially expressed genes (DEGs) and differentially expressed proteins (DEPs) in *R. soongorica* leaves treated with 0, 200, and 500 mM NaCl for 72 h. The results indicated that compared with the 0 mM NaCl treatment group, 2391 and 6400 DEGs were identified in the 200 and 500 mM NaCl treatment groups, respectively, while 47 and 177 DEPs were also identified. Transcriptome and proteome association analysis was further performed on *R. soongorica* leaves in the 0/500 mM NaCl treatment group, and 32 genes with consistent mRNA and protein expression trends were identified. *SYP71*, *CS*, *PCC13-62*, *PASN*, *ZIFL1*, *CHS2*, and other differential genes are involved in photosynthesis, vesicle transport, auxin transport, and other functions of plants, and might play a key role in the salt tolerance of *R. soongorica*. In this study, transcriptome and proteome association techniques were used to screen candidate genes associated with salt tolerance in *R. soongorica*, which provides an important theoretical basis for understanding the molecular mechanism of salt tolerance in *R. soongorica* and breeding high-quality germplasm resources.

## 1. Introduction

Due to climate change and human activities, soil salinization in arid and semiarid regions has become increasingly severe, which has become a major obstacle to the high-quality development of the ecological environment and economy in these regions [1]. Excessive accumulation of soil salts inhibits plant growth, reduces species diversity, changes the composition of plant communities, and reduces the biological productivity and biomass of the ecosystem [2,3]. Plants have a series of tolerance mechanisms to cope with salt stress, including modulation of various morpho-physiological attributes, antioxidant machinery, osmotic balance, and phytohormones [4]. Farhangi et al. [5] found that the body of *Phaseolus vulgaris* L. accumulates a large amount of soluble sugars to alleviate salt stress damage to cells. Glucose and sucrose in soluble sugars play important roles in plant growth and development and may be involved in regulating plant response to salt as signaling substances [6]. In addition, recent findings have shown that STI is a suitable tolerance index that can evaluate the salt tolerance of plants at different salt concentrations and determine the salt threshold concentration of plants [7,8]. Therefore, it is of great significance to understand the perception and response of plants to salt stress in order to improve the salt tolerance of plants.

The response of plants to abiotic stress involves a complete set of fine expression regulation mechanisms, such as transcriptional regulation, post-transcriptional regulation, translational regulation, and post-translational regulation [9]. The genomic resources of plants growing under salt stress provide important benchmarks for improving plant salt tolerance, alleviating salt damage, and improving soils [10,11]. Aliakbari et al. [12] analyzed the gene expression pattern of *Salicornia persica* using RNA-Seq technology and identified 1595 differentially expressed genes under salinity. Functional annotation analysis showed that energy homeostasis and primary metabolite synthesis play key roles in salinity adaptation. Chen et al. [13] investigated the leaf proteome of *Apocynum venetum* L. under salt stress and found that differential proteins were mainly involved in carbohydrate and energy metabolism, metabolite biosynthesis, and signal transduction. Dehydrin 1, annexin, pathogenesis-related protein, and peroxidase were also identified. Zhang et al. [14] used transcriptional sequencing and isobaric tag for relative and absolute quantitation (iTRAQ) proteomics analysis to identify 5432 genes and 43 proteins differentially expressed in *Helianthus tuberosus* L. leaves under salt stress, and these genes were mainly enriched in carbohydrate metabolism, ribosome activation and translation, redox, and ion binding. Transcriptome and proteome association found that the induced activity of ribosome and sugar signaling may confer *Helianthus tuberosus* L. with salt tolerance. Therefore, transcriptome and proteome techniques can be used as an effective method to screen potential salt-tolerance genes in plants. Since transcriptome and proteome reflect gene expression at different levels, it is necessary to detect mRNA and protein expression levels and perform omics data integration analysis to comprehensively explore the complex life activities of organisms and lay the foundation for revealing the regulatory rules of complex life activities of organisms at the molecular level.

*Reaumuria soongorica* is a super xerophytic salt-tolerant small shrub. It is a typical construction species and dominant species in the ecosystem of arid and semiarid regions, with strong stress resistance, grazing tolerance, sand-collecting ability, etc. [15]. Its leaves are rich in proteins, fats, and trace elements. It is the main vegetation for the construction of fodder bushes and the cultivation of degraded grasslands [16]. The unique salt gland structure of *R. soongorica* is an essential guarantee for its survival in saline environments. Its primary function is to transport salt secreted by the plant from the surrounding tissues to large vesicles that collect the salt. Subsequently, the substances accumulated by the secretory cells are transported into their small vesicles. These are constantly moving towards the cell membrane side and are finally excreted through the outer plasma membrane of the secretory cells, thus improving their osmotic adjustment ability and maintaining normal plant growth under salt stress [15]. At present, a number of research teams have conducted physiological and biochemical studies on *R. soongorica* seedlings under salt stress, mainly focusing on ion absorption, seed germination, and the antioxidant capacity of callus [17,18,19,20,21]. However, there are few studies on the molecular mechanism of salt tolerance of *R. soongorica* seedlings [9,22]. The study of integrated transcriptome and proteome analysis of the response to salt stress has not been reported. In this study, modern biological techniques were used to investigate the differentially expressed genes and protein expression profiles of *R. soongorica* responding to salt stress, the key genes and proteins involved in the salt tolerance defense system were explored, and the regulatory mechanism of *R. soongorica* responding to salt stress was elucidated. This study not only provided a new understanding of the molecular response mechanism of *R. soongorica* to salt stress but also provided potential genetic resources for breeding *R. soongorica*.

## 2. Results

### 2.1. Effects of NaCl Concentrations on Growth and Development of R. soongorica Seedling

Under the same stress treatment time, the plant height of *R. soongorica* first increased and then decreased with increasing salt concentration. Compared with the control at 24 h of stress treatments, the plant height of all NaCl treatments showed no significant difference. At 72, 144, and 216 h of stress treatments, plant height was highest under the 200 mM NaCl concentration, and plant height was the lowest under the 500 mM NaCl concentration. In the same salt concentration treatment, plant height showed an increasing trend with increasing days of stress. Compared to 24 h of stress, plant height increased by 36.62%, 37.67%, 44.43%, 28.45%, 17.59%, and 13.26% under different concentration treatments at 216 h of stress (Table 1). Furthermore, it can be seen from Table 1 that the root length of *R. soongorica* seedlings first increased and then decreased with increasing NaCl treatment concentration. At 24 h and 72 h of stress, the root length under the 200 mM NaCl treatment increased by 6.98% and 4.09% compared with the control, respectively. At 144 h and 216 h of stress, the root length gradually decreased with increasing NaCl concentration.

**Figure 1:**
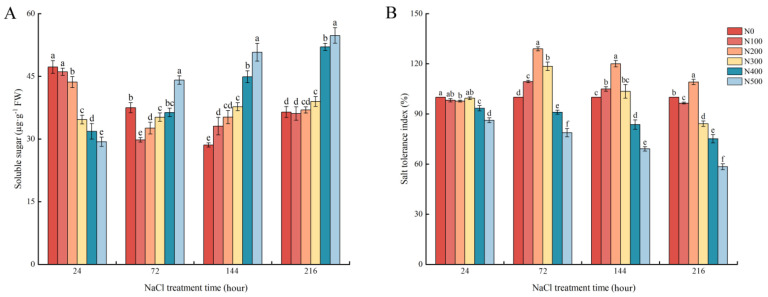
Physiological changes in *R. soongorica* leaves under salt stress. (**A**) Effect of NaCl stress on the soluble sugar content of *R. soongorica* leaves. (**B**) Changes in salt tolerance coefficient of *R. soongorica* under NaCl stress. N0: 0 mM NaCl; N100: 100 mM NaCl; N200: 200 mM NaCl; N300: 300 mM NaCl; N400: 400 mM NaCl; N500: 500 mM NaCl. Different lowercase letters indicate significant differences from different salt levels (*p* < 0.05).

Figure 1A shows that different NaCl treatment concentrations significantly changed the soluble sugar content in *R. soongorica* leaves. The soluble sugar content decreased with the increase in NaCl treatment concentration at 24 h of treatment, and the soluble sugar content showed a trend of first increasing and then decreasing with the increase in NaCl treatment concentration at 72 h of treatment. The soluble sugar content gradually increased with the increase in NaCl treatment concentration at 144 and 216 h of treatment. The soluble sugar content seemed to increase at different rates during the whole treatment cycle, especially when the soluble sugar content in leaves increased to the maximum after 144 h of the 500 mM NaCl treatment and then remained at a high level. Figure 1B shows that the tolerance index of *R. soongorica* seedlings to NaCl was significantly lower than that of other treatments at 400 and 500 mM NaCl for 24 h. Except for the 24 h NaCl treatment, the tolerance index of seedlings to NaCl first increased and then decreased with increasing NaCl concentration at the other treatment time. The tolerance index of seedlings was the highest under the 200 mM NaCl treatment, and then it started to decrease gradually. With the prolongation of salt stress time, the tolerance index of seedlings was gradually decreased under the 400 and 500 mM NaCl treatments. When the treatment concentration reached 500 mM NaCl after 216 h of NaCl treatment, the tolerance index of the seedlings was only 58.42%.

### 2.2. De Novo Assembly and Annotation of the R. soongorica Transcriptome

**Figure 2:**
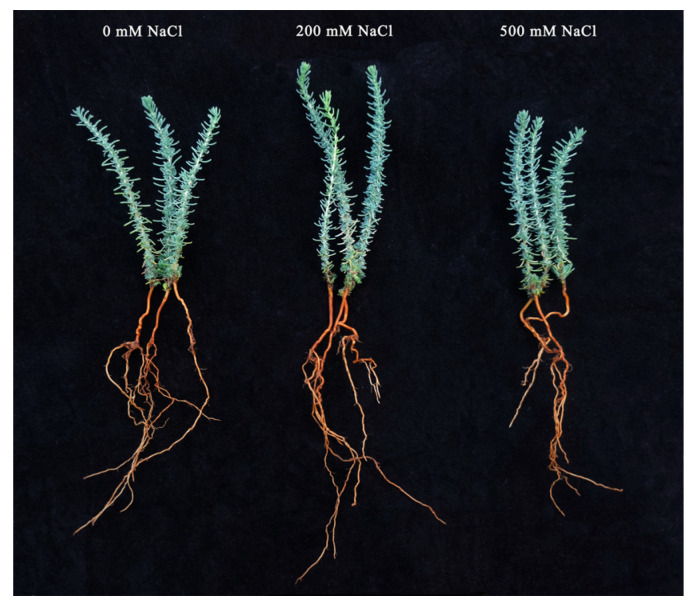
A representative figure of the phenotypic differences of *R. soongorica* seedlings under different treatments.

**Figure 3:**
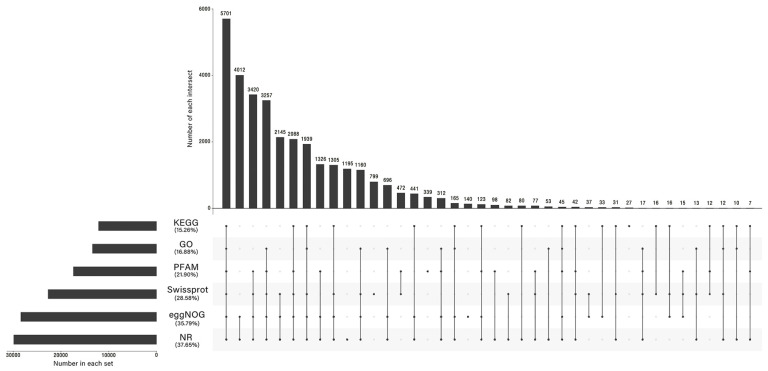
Annotation statistics of Unigene in *R. soongorica* leaves under NaCl stress.

To comprehensively understand the transcriptomic profiles of *R. soongorica* and to identify differentially expressed genes (DEGs) in response to salt stress, we sequenced the transcriptomes of nine different *R. soongorica* libraries, and (A1, A2, A3), (B1, B2, B3), and (C1, C2, C3) were three biological replicates of 0 mM NaCl (control), 200 mM NaCl, and 500 mM NaCl, respectively (Figure 2). First, total RNA was extracted from the 0 mM NaCl (control), 200 mM NaCl, and 500 mM NaCl-treated samples, and RNA sequencing was performed by Illumina Hiseq. A total of 388,789,330 clean reads were obtained from nine samples using low-quality (Q-value < 20) and multiple N-base filtered reads (Table S3). The filtered clean reads were assembled using Trinity 2.5.1 software and the longest transcripts were selected as unigenes. After isoform detection, a total of 79,307 unigenes longer than 300 bp were obtained from these nine libraries. All single gene sequences were identified using Blastx and compared with NR (NCBI non-redundant protein sequences), GO, KEGG, egg NOG (Evolutionary genealogy of Genes: Non-supervised Orthologous Groups), Swiss-Prot and Pfam databases, and the annotation rates were 37.65%, 16.88%, 15.26%, 21.90%, 35.79%, and 28.58%, respectively (Figure 3).

### 2.3. Transcriptome Data Profiling of R. soongorica Leaves

**Figure 4:**
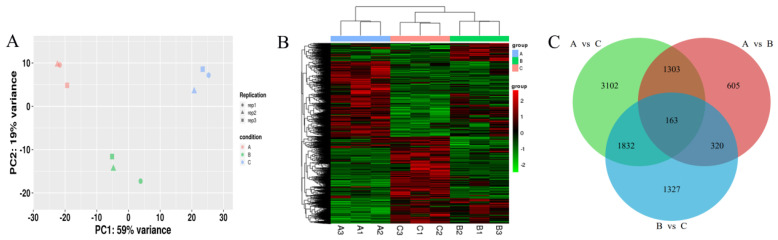
Identification and analysis of DEGs in *R. soongorica* leaves under 0, 200, and 500 mM NaCl. (**A**) The similarity of gene expression was compared in three-sample groups using PCA. (**B**) Cluster analysis of DEGs in *R. soongorica* leaves under different NaCl treatments. (**C**) Common or unique DEGs are compared using the Venn diagram.

The expression levels of the same gene in different samples and the expression patterns of DEGs in the same sample are based on RNA-Seq data. A heat map was constructed based on the Euclidean method to calculate distances and the hierarchical clustering longest distance method (complete linkage) to analyze quantitative differences in the expression levels of DEGs in all comparison groups (Figure 4B). The cluster analysis results showed significant differences between the control and the salt-treated groups. In addition, principal component analysis (PCA) was performed on each sample according to the expression level (Figure 4A). Significant differences were found between the transcriptomes of *R. soongorica* leaves under different salt treatments, and the data were well reproducible within groups. Statistical results of DEGs between the 0, 200, and 500 mM NaCl treatment groups are shown in Figure 4C: compared with the 0 mM NaCl treatment, under the 200 mM NaCl treatment, i.e., 2391 (1057 up-regulated/1334 down-regulated) DEGs, compared with the 500 mM NaCl treatment, i.e., 6400 (3093 up-regulated/3307 down-regulated) DEGs, and the 500 mM NaCl treatment compared to the 200 mM NaCl treatment, i.e., 3642 (2169 up-regulated/1473 down-regulated) DEGs (Table S4). The comprehensive results showed that there were more down-regulated genes than up-regulated genes under the salinity treatment compared to the 0 mM NaCl treatment, while the number of DEGs of *R. soongorica* leaves treated with high NaCl concentrations was significantly higher than that of leaves treated with low NaCl concentrations.

**Figure 5:**
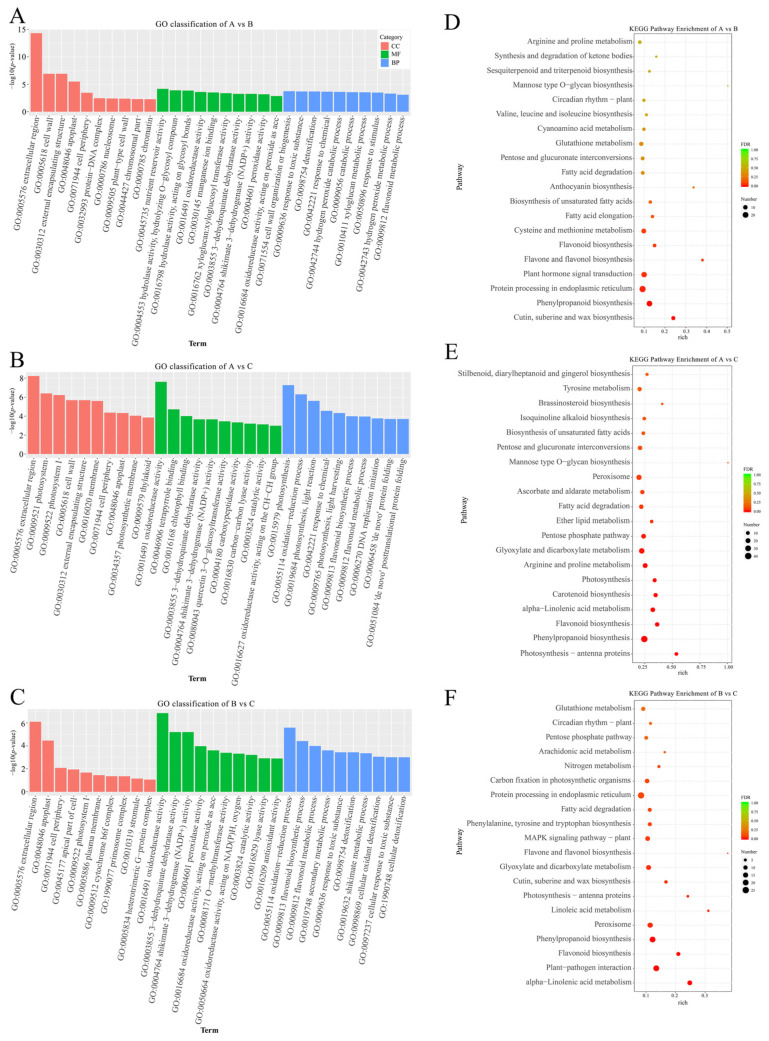
GO classification and KEGG pathway of DEGs under different salt concentration stresses. (**A**–**C**) GO classification of DEGs in *R. soongorica* leaves under different NaCl treatments. (**D**–**F**) KEGG pathway enrichment of DEGs in *R. soongorica* leaves under different NaCl treatments.

GO enrichment analysis was performed on the DEGs treated with different concentrations of NaCl, and GO classification was performed according to molecular function, biological process, and cellular component. The top 10 GO terms with the most significant enrichment in each GO classification were selected for display, and the results are shown in Figure 5A,B. Compared with the 0 mM NaCl treatment, DEGs in the 200 mM and 500 mM NaCl treatment groups were significantly enriched in the extracellular region, cell wall, photosystem, oxidoreductase and peroxidase activity, xyloglucan metabolic process, flavonoid metabolic process, etc. Compared with the 200 mM NaCl treatment group, DEGs in the 500 mM NaCl treatment group were significantly enriched in the apoplast, 3-dehydroquinate dehydratase activity, dioxide-reduction process, flavonoid biosynthesis process, and so on (Figure 5C). In addition, KEGG enrichment analysis showed that DEGs in mannose-type O-glycan biosynthesis, anthocyanin biosynthesis, flavone and flavonol biosynthesis, photosynthesis-antenna proteins, brassinosteroid biosynthesis, etc., were significantly enriched after the 200 mM and 500 mM NaCl treatments compared with the 0 mM NaCl treatment (Figure 5D,E). Comparing the 500 mM and 200 mM NaCl treatment groups, DEGs in flavone and flavonol biosynthesis, linoleic acid metabolism, festoon antenna proteins, and other pathways were significantly enriched (Figure 5F). These results suggest that *R. soongorica* leaves respond to salt stress primarily by regulating cellular metabolism and photosynthesis.

### 2.4. Proteomic Data Profiling of R. soongorica Leaves

**Figure 6:**
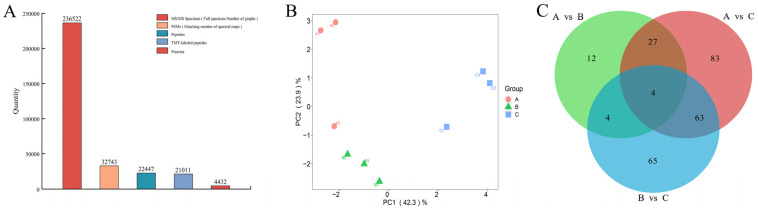
Identification and analysis of DEPs in *R. soongorica* leaves under 0, 200, and 500 mM NaCl. (**A**) Protein information identified by TMT. (**B**) The similarity of protein expression was compared in three sample groups using PCA. (**C**) Common or unique DEPs are compared using the Venn diagram.

A total of 236,522 chromatograms were obtained from the mass spectrometry experiment. After analysis by Proteome Discoverer 2.2 software, 32,743 chromatograms were matched: 4432 proteins and 22,447 peptides were identified, including 21,011 TMT-labeled peptides. The peptide labeling efficiency was 93.6% (Figure 6A). PCA shows that PC1, which explains 42.3% of the total variation, cleanly separates plants from the 200 mM NaCl treatment from those from the 500 mM and 0 mM NaCl treatments, and the data within the group have good repeatability. PC2, which explained 23.9% of the total variation, showed differences between the 0 mM and 500 mM NaCl treatments (Figure 6B). Statistical results of differentially expressed proteins (DEPs) between the 0, 200, and 500 mM NaCl treatments are shown in Figure 6C. Comparison of salt-treated plants with the 0 mM NaCl treatment revealed differential expression of 47 (36 up-regulated/11 down-regulated) and 177 (126 up-regulated/51 down-regulated) DEPs at 200 and 500 mM NaCl treatment, respectively. Compared to the 200 mM NaCl treatment, 69 up-regulated and 67 down-regulated proteins were recorded in the 500 mM NaCl treatment (Table S5). By analyzing different DEPs, it was found that the number of DEPs induced by 500 mM NaCl was significantly higher than that induced by 200 mM NaCl compared with the 0 mM NaCl treatment.

**Figure 7:**
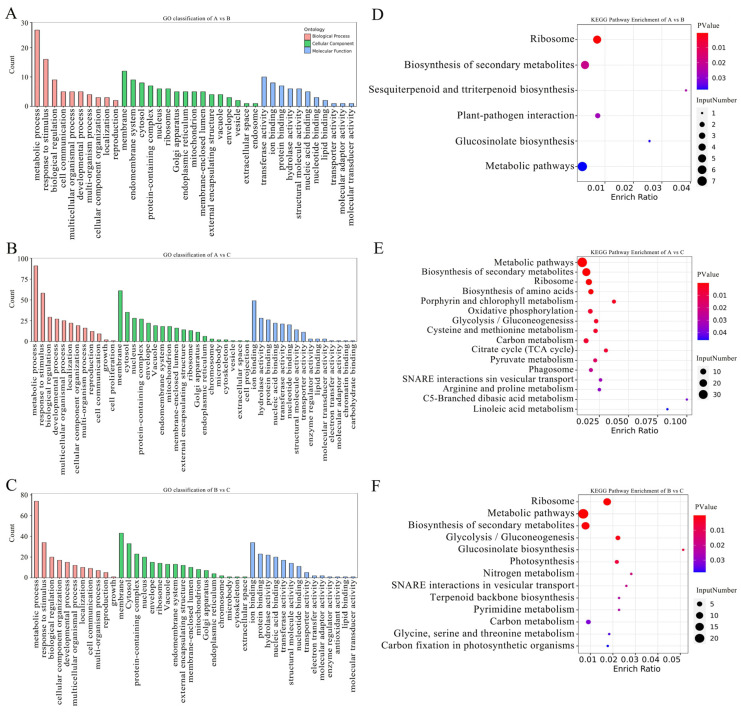
GO classification and KEGG pathway of DEPs under different salt concentration stresses. (**A**–**C**) GO classification of DEPs in *R. soongorica* leaves under different NaCl treatments. (**D**–**F**) KEGG pathway enrichment of DEPsin *R. soongorica* leaves under different NaCl treatments.

GO functional enrichment analysis of the DEPs in the three comparison groups showed that there were 10, 12, and 11 significant enrichment items (*p* < 0.05) in the biological processes of the 0/200, 0/500, and 200/500 comparison groups, respectively (Figure 7A–C). They mainly included metabolic processes, response to osmotic stress, biological regulation, etc. In the 0/200 comparison group (Figure 7A), 11 items were enriched in molecular function, mainly involving transferase activity, ion binding, protein binding, etc. There were 16 items enriched in cellular component, mainly involving membrane, endomembrane system, cytosol, etc. In the 0/500 comparison group (Figure 7B), 11 items were enriched in molecular function, mainly involving ion binding, hydrolase activity, protein binding, etc. There were 16 items enriched in cellular component, mainly involving membrane, cytosol, nucleus, etc. In the 200/500 comparison group (Figure 7C), 14 items were enriched in molecular function, mainly involving ion binding, protein binding, hydrolase activity, etc. There were 17 items enriched in cellular component, including membrane, cytosol, and protein-containing complex. According to the above data, the DEPs of *R. soongorica* leaves respond to a variety of biological functions under salt treatment.

To further understand the biological functions of the proteins, KEGG enrichment analysis was performed on the annotated DEPs. The results showed that compared with 0 mM NaCl treatment, the DEPs in the 200 mM NaCl treatment group were significantly enriched in sesquiterpenoid and triterpenoid biosynthesis, glucosinolate biosynthesis, and other pathways (Figure 7D). In the 500 mM NaCl treatment group, DEPs were significantly enriched in linoleic acid metabolism, porphyrin, chlorophyll metabolism, and other pathways (Figure 7E). Compared with the 200 mM treatment group, DEPs in the 500 mM NaCl treatment group were significantly enriched in glucosinolate biosynthesis, nitrogen metabolism, SNARE interactions in vesicular transport, and other pathways (Figure 7F).

### 2.5. Transcriptomic and Proteomic Association Analysis

**Figure 8:**
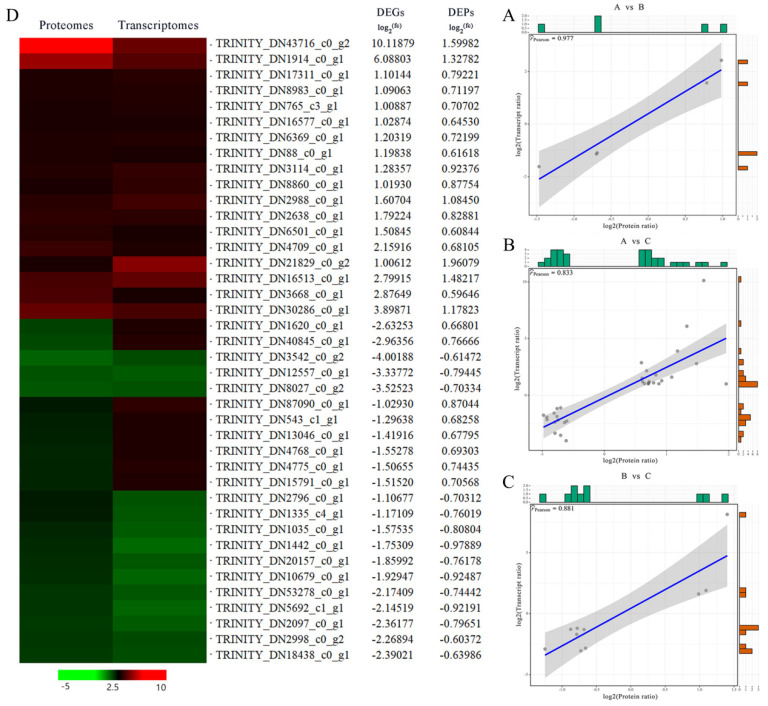
Correlation analysis based on proteomics and transcriptome. (**A**–**C**) Correlation analysis plots for different concentrations of NaCl comparison groups, with DEP expression levels labeled on the horizontal axis and DEG expression levels labeled on the vertical axis. (**D**) Expression pattern clustering analysis of 40 differentially expressed mRNAs and proteins. fc: fold of change. Red represents up-regulated and green represents down-regulated.

To correlate transcript and protein expression profiles, accession numbers were extracted from the proteome and compared to annotated RNA-Seq libraries (Table S6). According to the association analysis, there were 5, 32, and 10 genes with the same protein and mRNA changes in the 0/200, 0/500, and 200/500 mM NaCl comparison groups, respectively. There were zero, eight, and two genes with opposite trends in protein and mRNA expression, respectively. The genes with differentially expressed proteins but no differentially expressed mRNA were 40, 118, and 122, respectively, indicating that only a few proteins were directly regulated at the transcriptional level. Meanwhile, correlation analysis was performed for the DEPs and DEGs with consistent expression levels in the three control groups, and the Pearson correlation coefficients (r) were 0.977 (Figure 8A), 0.833 (Figure 8B), and 0.881 (Figure 8C), respectively. Further analysis of the differentially expressed genes screened from the 0/500 mM NaCl treatment group (Figure 8D) showed that 18 of the 32 genes with consistent mRNA and protein expression trends were up-regulated and 14 were down-regulated (Table 2).

Using the KEGG pathway database, pathway enrichment analysis was performed on the DEPs with the same gene expression trend in the 0/500 comparison group to identify the major metabolic and signaling pathways involved in the proteins. The results showed that 13 of the 32 DEPs were distributed over 19 pathways (Table 3). The DEPs were involved in cysteine and methionine metabolism, citrate cycle (TCA cycle), SNARE interactions in vesicular transport and photosynthesis, etc. Through the above association analysis, some target genes that might be related to the salt stress response of *R. soongorica* were screened out, such as *SYP71*, *CS*, *PCC13-62*, *PASN*, *ZIFL1*, and *CHS2* (Table 4). These differential genes may play a key role in the molecular mechanism of salt stress response in *R. soongorica*.

## 3. Materials and Methods

### 3.1. Experimental Materials and Treatment

The research subjects were *R. soongorica* seeds collected from natural growing sites in Laohukou, Wuwei, Gansu, China (102°58′ E, 38°44′ N) at the end of October 2019. The seeds were collected according to the Technical Regulations for Seed Collection of Rare and Endangered Wild Plants (LYT 2590-2016) and stored in a storage cabinet (CZ-250FC, Top Yunong, Zhejiang, China) for later use. The present study was conducted by pot culture in 2020 at the Experimental Station of Longqu Seed Orchard, Gansu Province Academy of Qilian Water Resource Conservation Forests Research, in Zhangye, Gansu, China. In April 2020, seeds of the same full size were selected, disinfected with 0.3% KMnO_4_ solution for 15 min, rinsed 5 times with deionized water, and planted in a plug tray with a diameter of 4.5 cm and a height of 8.5 cm. The culture medium was vegetative soil, quartz sand, and vermiculite (3:1:1), and three seeds were planted in each plug tray. The seedlings were then grown in a greenhouse at 25 ± 1 °C, 50% humidity, natural ventilation, and good lighting. They were regularly irrigated with groundwater. On 15 June 2020, the uniformly sized *R. soongorica* seedlings were carefully removed from the plug trays, transferred to plastic pots (20 × 23 × 25 cm) with 2.5 kg of soil, and then grown in the greenhouse. The available phosphorus, salinity, and pH of the tested soil were 26.6 mg/kg, 0.2%, and 8.3, respectively. The smart irrigation control systems were used to maintain the soil water content close to the field capacity (60%). The *R. soongorica* seedlings were planted in a total of 1000 pots (4 seedlings per pot). After growing the seedlings for 40 d, 720 pots of seedlings with relatively uniform growth were selected as experimental material. The *R. soongorica* plants were subjected to the following six salt treatments: NaCl concentrations of 0, 100, 200, 300, 400, and 500 (mM). Four blocks of the same NaCl treatment were performed (morphological, physiological, transcriptomic, proteomic) and each NaCl treatment concentration was replicated on 10 pots per and three biological replicates were performed, using a total of 180 pots per block.

According to the experimental design, the corresponding NaCl solution was prepared with deionized water, and the NaCl solution was poured evenly around the root of *R. soongorica* with a syringe (to make sure that there was no permeability phenomenon when the prepared NaCl solutions were completely poured into the flowerpot). In order to avoid osmotic shock caused by salt shock reaction, the target concentration was reached within 24 h by gradual application of salt. Corresponding indices were determined after NaCl treatment for 24, 72, 144, and 216 h, respectively. A total of 5 g of leaves from each treatment was rapidly frozen in liquid nitrogen and then stored in an ultra-low temperature refrigerator (−80 °C) for proteome and transcriptome determination.

### 3.2. Determination of Morphological and Physiological Indicators

After 24, 72, 144, and 216 h of salt treatment, 3 seedlings were randomly selected from each replicate of each treatment, and a total of 9 seedlings were gently straightened. Plant height and root length were measured with a ruler (measuring range: 20 cm, accuracy: 0.1 cm) and averaged.

The soluble sugar content of *R. soongorica* leaves was determined using the methods described by Tan et al. [23]. Briefly, 0.1 g of *R. soongorica* leaves were weighed and placed in a 20 mL glass tube with a stopper, 10 mL of distilled water was added, the extract was extracted in boiling water for 30 min, the extract was filtered into a 25 mL volumetric flask, and the volume was kept constant. A total of 0.5 mL of the sample extract was absorbed into a 20 mL scale tube; 1.5 mL of distilled water was added; 0.5 mL of anthrone ethyl acetate reagent and 5 mL of concentrated sulfuric acid were added to the tube, shaken well, and cooled to room temperature, and the absorbance value was measured at a wavelength of 630 nm.

$$\mathrm{Soluble}\;\mathrm{sugar}\;\mathrm{content}\;(\mu g\cdot g^{-1})=\frac{C\cdot V\cdot N}{V_{t}\cdot W}\times 100\%$$

where C represents the glucose content determined from the standard curve (µg); V is the total volume of the extract (mL); N is the dilution ratio; $V_{t}$ represents the amount of sample added during the determination (mL); W represents the fresh weight of the sample (g).

The salt tolerance index (STI) was calculated according to the method of Roshdy et al. [24]. The formula is as follows:

$$\mathrm{STI}=\left({\mathrm{DW}}_{\mathrm{Nacl}}/{\mathrm{DW}}_{\mathrm{contrast}}\right)\times 100\%$$

where ${\mathrm{DW}}_{\mathrm{NaCl}}$ represents the dry weight of plants under salt treatment (g); ${\mathrm{DW}}_{\mathrm{contrast}}$ represents the dry weight of control plants (g).

### 3.3. RNA Sample Preparation and Transcriptome Analyses

Differential gene analysis and identification of *R. soongorica* leaves was performed according to the method described by Anders et al. [25] with some modifications. Briefly, total RNA was extracted from the collected 50 mg of *R. soongorica* leaves using the plant RNA purification kit (Norgen, Thorold, ON, Canada) according to the manufacturer’s instructions. The quality of total RNA was then measured using an Agilent 2100 Bioanalyzer (Agilent, Santa Clara, CA, USA). Oligo (dT) magnetic beads (Biomag, Wuxi, China) were used to enrich mRNA with PolyA structure in total RNA, and the mRNA was cleaved into 200−300 bp fragments by ion disruption. Using the RNA as a template, the first strand cDNA was synthesized using 6-base random primers (Gdsbio, Guangzhou, China) and reverse transcriptase (Aidlab, Beijing, China). The first strand cDNA was used as a template to synthesize the second strand cDNA, and the library size was 300–400 bp. Quality control was performed by Agilent 2100 Bioanalyzer, and double-terminal sequencing was performed by Illumina HiSeqTM2000 (NGS Solexa Hiseq2000, Illumina, CA, USA). All the above tests were performed by Suzhou Panomico Biotechnology Co., LTD.

RNA-Seq raw sequencing data were converted by invoking Base, followed by quality control of the raw reads. Cutadapt 1.16 software was used to remove the original data to obtain clean reads, and Trinity 2.5.1 software (Broad Institute, Hebrew University of Jerusalem, Jerusalem, IL) was used to splice clean reads to obtain transcripts. The longest transcript under each gene is extracted as the representative sequence of the gene after splicing, called the unigene. The unigene was used as the reference sequence for subsequent analysis. Clean reads for each gene were calculated and normalized to reads per kilobase per million reads (RPKM) for gene expression analysis. Differential analysis of gene expression was performed by DESeq screening for differentially expressed genes with the following conditions: multiple expression differences |log2FoldChange| > 1, significance *p*-value < 0.05.

Meanwhile, to verify the reliability of the transcriptome data, we randomly selected 10 differential genes with significant changes in expression for quantitative real-time PCR (qRT-PCR) analysis in the A vs. B and A vs. C comparison groups, respectively. Specific primers were designed using Primer 3.0 software (Table S1) and synthesized by Sangon Biotech (Shanghai) Co., Ltd. The internal reference gene used was “DN11735_c0_g2”. Sample RNA was extracted using the Plant Total RNA Extraction Kit (DP-437) (Tiangen, Beijing, China). cDNA synthesis was performed using the PrimeScript™ RT Master Mix (Perfect Real Time) Kit (TaKaRa, Dalian, China), and the instructions for experimental procedures were followed. The qRT-PCR program was 95 °C for 30 s, 95 °C for 5 s, 60 °C for 30 s, 95 °C for 5 s, 60 °C for 60 s, and 50 °C for 30 s, for a total of 40 cycles. The experiment was subjected to three biological replications. The relative expression of each gene was calculated using the 2^−ΔΔCt^ method. The results showed that the expression trends of the 20 differential genes selected from *R. soongorica* leaves were highly correlated with the RNA-Seq results (Table S2). This indicates that the transcriptome data are reliable.

### 3.4. Protein Sample Preparation and Proteomic Analysis

Proteins were extracted from 2 g of *R. soongorica* leaves according to the method of Chen et al. [26], protein quality was detected by sodium dodecyl sulfate polyacrylamide gel electrophoresis (SDS-PAGE), and protein samples were stored in a refrigerator at −80 °C until use. The tandem mass tag (TMT) assay was performed by Suzhou Panomic Biotechnology Co., LTD. according to the manufacturer’s recommendations (Applied Biosystems, Foster City, CA, USA). Briefly, 200 mL of protein lysates were taken from each sample for typing digestion, and then the peptides were labeled by TMT. The labeled groups were mixed and the mixed peptides were pre-separated by strong cation exchange chromatography. The liquid phase was separated on a SCX column after vacuum drying. Then, mass spectrometry (LC-MS/MS) (Applied Protein Technology, Shanghai, China) based on Orbitrap Fusion Lumos (Thermo Fisher Scientific, Waltham, MA, USA) was performed. Peak identification was performed on the original documents of mass spectrometry to obtain the peak list, and then the reference database was established, and the peptides and proteins were identified. In this study, the screening conditions of differentially expressed proteins were as follows: when the protein difference multiple was >1.5 or <0.66, the significance *p*-value < 0.05 was used as the screening condition for differential proteins.

### 3.5. Statistical and Bioinformatic Analysis

Statistical analyses were performed using SPSS 19 software. All data are expressed as mean ± standard error (SE) of three independent replicates. Significant mean differences between treatments were performed by one-way analysis of variance based on Duncan’s multiple range test at the level of *p* ≤ 0.05. Blast2go 2.5.0 software was used for Gene Ontology (GO) annotation analysis of the identified differential genes/proteins. During the analysis, the gene list and gene number of each term were calculated using the differential genes annotated by the GO term. Then, the *p*-value was calculated by the hypergeometric distribution method. The threshold for significant enrichment was set at *p*-value < 0.05. Kobas 3.0 software was used for pathway enrichment analysis of the identified genes/proteins in the Kyoto Encyclopedia of Genes and Genomes (KEGG) pathway database, with a *p*-value < 0.05 as the screening criterion for significant enrichment. Blast comparison was performed between the identified protein sequences and the GO and KEGG background databases, respectively. *Arabidopsis* was confirmed as the species with the best comparison results, and the protein information of the mutual comparison was determined. Therefore, the comparative protein information of *Arabidopsis* was used for the subsequent functional enrichment analysis. The expression patterns of DEPs and DEGs were hierarchically clustered using the MultiExperiment Viewer (MeV) software version 4.9.0 [27]. The relative ratios of DEPs and DEGs were subjected to log_2_ transformation, the Euclidean distance similarity metric was used to define similarity, and hierarchical clusters were assembled using the complete linkage clustering method. The clustering results were visualized by MeV 4.9.0 software.

### 3.6. Integrative Transcriptome-Proteome Analysis

The integrated transcriptome-proteome analysis calculates Pearson correlation coefficients (r) from the fold change of expressed transcripts and proteins to assess the correlation between the expression levels of specific transcripts and proteins in the transcriptome and proteome profiles of the samples [28]. 

## 4. Discussion

The occurrence of soil salinization has been further exacerbated by changes in climatic conditions, such as global warming and increased drought [29]. As one of the most detrimental factors among abiotic stresses, salt stress has disrupted normal physiological metabolic processes in plants, causing severe growth dysfunction in photosynthesis, protein synthesis, and energy metabolism [30]. Meanwhile, the maintenance of plant growth is directly related to the salt tolerance of plants [31]. In saline environments, halophytes can employ a variety of morphological and physiological adaptation strategies to reduce the excessive production of salt ions [32]. For example, a study of *Halogeton glomeratus* found that its optimal growth occurred under conditions of approximately 100 mM NaCl; however, it began to decline at higher salinities [11]. Rahman et al. [33] reported that salinity significantly reduced the growth and development of *Achras sapota*, which was accompanied by a significant decrease in plant height, root length, and plant STI. In the present study, after 72 h of NaCl stress, the plant height and root length of *R. soongorica* seedlings first increased and then decreased with increasing NaCl concentration, and the highest values were found at a 200 mM NaCl concentration. However, when the NaCl concentration exceeded 200 mM, plant height and root length decreased with increasing NaCl concentration. This result is consistent with the findings of Wang et al. [11], but different from those of Rahman et al. [33]. This difference in results is most likely due to the different salt-adaptation strategies of the species. First of all, *R. soongorica* and *Halogeton glomeratus* have been identified as salt-tolerant plants and the growing areas are mainly distributed in northwestern China [11,19], while *Achras sapota* is mainly a fruit tree native to Central America and Mexico [33]. At the same time, the main salt-adaptation mechanism in *H. glomeratus* is the translocation of sodium and some toxic ions into specific salt storage cells in the leaves, which is the same role of the specially organized salt glands in the leaves of *R. soongorica* [19]. In contrast, the salt-adaptation strategy of *A. sapota* is to retain sodium ions in the roots and accumulate compatible solutes to mitigate salt-toxic effects. Finally, it was found that both *R. soongorica* and *H. glomeratus* possessed good salt-adapted growth at less than 200 mM NaCl stress based on the data, while *A. sapota* showed a positive correlation of growth indicators with concentration when based on salt stress. At the same time, this study found that when treated with NaCl for 24 h, the soluble sugar content gradually decreased with increasing treatment concentration. When treated with NaCl for 72 h, the soluble sugar content gradually increased with increasing treatment concentration, had the maximum increase after 144 h, and then remained at a high level. This is consistent with the changes in soluble sugar content in *R. soongorica* leaves after different times and concentrations of NaCl treatment by Yang et al. [34], which may be due to the fact that soluble sugar was mainly used as an energy source to ensure the normal growth of *R. soongorica* in the early stages of NaCl treatment. With the prolongation of NaCl treatment time, the intracellular ion content increased, and soluble sugar was mainly used as an osmotic adjustment substance to maintain the osmotic balance of cells to ensure the growth of cells under salt stress.

In this study, the trends of gene expression and protein levels in *R. soongorica* leaves under different salt treatments (0, 200, and 500 mM NaCl) were analyzed. More differential genes and proteins were found in the 0/500 compared group. Meanwhile, the results of DEG and DEP analysis showed that the number of down-regulated genes was greater than that of up-regulated genes, while slightly more up-regulated proteins were identified.

Through association analysis of transcriptomic and proteomic data, the internal relationship between genes and proteins can be deeply understood, which is of great significance in mining reliable genes for plant breeding and improvement [35]. Jiang et al. [27] performed correlation analysis on the transcriptome and proteome of cucumber seedlings treated with H_2_S under salt stress. The results showed that the correlation coefficient of differentially expressed proteins and genes with the same trend of change under H_2_S treatment was 0.839, and most of the associated differential proteins were enriched in photosynthesis, cysteine metabolism, and energy metabolism. Ding et al. [36] analyzed tomato (*Solanum lycopersicum*) leaves under stress conditions by combining transcriptome and proteome and found 79 differential proteins with the same expression trend of the transcriptome, most of which were related to stress response and protein folding. In this study, transcriptome and proteome association analysis was performed on three comparison groups (0/200, 200/500, and 0/500) of *R. soongorica* leaves after 72 h of NaCl stress. After screening, only a small number of *R. soongorica* DEPs are consistent with the mRNA expression level. Correlation analysis revealed that DEPs and DEGs with consistent expression levels in the three control groups had a Pearson correlation > 8. This is because the transcription of DNA into mRNA and the translation of mRNA into proteins are affected by a variety of transcriptional, translational, and post-translational factors, including changes in the amount of transcribed mRNA and in the amount and function of proteins. This indicates that only a few proteins are directly regulated at the transcriptional level. Thus, the screened *R. soongorica* DEPs and DEGs showed a strong positive correlation with each other based on the number of their expression levels and their similar expression. Further analysis revealed that the number of genes with consistent transcriptome and proteome expression was significantly higher in the 0/500 comparison group (32) than in the 0/200 comparison group (5) and the 200/500 comparison group (10). Therefore, the 0/500 comparison group was focused as a study and further study revealed 32 differential proteins distributed in 19 metabolic pathways. It mainly involves cysteine and methionine metabolism, TCA cycle, SNARE interactions in vesicular transport, metabolism, etc. This further showed that *SYP71*, *CS*, *ZIFL1*, *PCC13-62*, *PASN*, and *CHS2* genes may play an important role in the molecular mechanism of *R. soongorica* response to salt stress.

SNARE factors are divided into Q-SNARE and R-SNARE [37]. When plants are exposed to adverse conditions, a large number of SNARE proteins are required to mediate the membrane fusion mechanism during vesicle trafficking to ensure the smooth closure of ion channels and transporters on the plasma membrane and the separation of harmful ions [38]. For example, the expression levels of R-SNARE VAMP7-type, QC-SNARE, and Qb/c-SNARE were significantly increased in tomatoes under salt stress [39], suggesting that the SNARE-mediated vesicle trafficking pathway plays an important role in the salt stress response. The SYP7 family is a plant-specific family of QC-SNARE proteins consisting mainly of SYP71, SYP72, and SYP73 homologous proteins. [40]. Rice showed stronger antioxidant and disease resistance by overexpressing the SYP71 protein [41]. In this study, it was found that the expression levels of the *SYP71* gene and protein were significantly up-regulated in *R. soongorica* leaves after the 500 mM NaCl treatment, suggesting that *SYP71* may be involved in the vesicle transport pathway in leaf cells and thus play an important role in the response to salt stress in *R. soongorica* leaves. Citrate synthase (CS) is the core enzyme of the mitochondrial TCA cycle and an organic acid that regulates the TCA cycle, which directly controls cellular function [42]. The adaptation of plant cells to salt stress is closely related to various metabolic processes [43]. *CS* can improve plant tolerance to saline-alkali soil [44]. Similar to NaCl-treated Zea mays [45] and *Haloxylon salicornicum* [46], high NaCl stress significantly increased the abundance of citrate synthase in *R. soongorica*. The abundance of the *CS* gene was also significantly increased in this study, indicating that *CS* is a key protein of *R. soongorica* in response to salt stress.

Salt stress in plants is regulated by many signaling molecules, and auxin is a key medium for plants to respond to salt stress [11], which plays an important role in plant development and salt stress. Studies have shown that salt stress significantly disrupts auxin homeostasis and distribution in primary roots and inhibits auxin signaling [47]. Auxin treatment can significantly restore the growth of *Arabidopsis*’s primary roots under salt stress [48]. The above results indicate that the distribution and signaling of auxin mediate the response of plants to salt stress. ABC and MFS are two families of transporters in the plant kingdom. Zinc-induced facilitator-like 1 (*ZIFL1*), a member of the MFS family, is critical for auxin transport [49]. Overexpression of *ZIF1* can enhance auxin transport and improve stress tolerance in *Arabidopsis* [50]. The dehydration-related protein PCC13-62 can improve plant tolerance to extreme drought [51]. Li et al. [52] found that the expression of PCC13-62 in upland cotton was up-regulated under salt stress based on iTRAQ proteomics techniques. In addition, Giarola et al. [53] also found that salt stress could activate the *PCC13-62* promoter and increase the tolerance of *Arabidopsis* to salt stress. The results of this study showed that after 500 mM NaCl treatment, the abundance of *ZIFL1* and *PCC13-62* significantly increased, suggesting that the leaves of *R. soongorica* could enhance the tolerance to salt stress by up-regulating the expression of *ZIFL1* and *PCC13-62*. Meanwhile, our study also found that the expression of *PSAN* in *R. soongorica* leaves decreased under salt stress. *PSAN* is a PSI-related gene, and salt stress can significantly reduce the photochemical activity of PSI, ultimately leading to lower photosynthetic efficiency [54]. This is consistent with the findings on photoinhibition of *Cinnamomum camphora* L. by NaCl stress [55], where salinity resulted in the down-regulation of protein expression located on PSI (*PSAN*). The expression of the *PASN* gene and protein in this study also showed the same trend of down-regulation. The results showed that *PSAN* may play a key role in the molecular mechanism of *R. soongorica*’s response to salt stress.

Polyketide synthases (PKSs) are a family of multifunctional proteins that exhibit remarkable versatility in structural fusion and functional organization to produce different classes of compounds. Structurally, chalcone synthase (CHS) is considered to be the simplest type III PKS. This enzyme is also known to catalyze the first step of the flavonoid/isoflavone pathway [56]. Flavonoids, an important secondary metabolite, are closely related to the antioxidant capacity of plants [13]. Since chalcone synthase is the first enzyme in the flavonoid biosynthetic pathway, its expression and regulation are important [57]. The significant enrichment of CHS protein in *Pongamia* roots under salt treatment contributes to the protection of *Pongamia* with high antioxidant activity from ROS damage and promotes root growth under high salt stress [58]. The results of this study showed that the abundance of *CHS2* was significantly increased after the 500 mM NaCl treatment, indicating that *R. soongorica* could be used to increase secondary metabolites to mitigate salt damage by up-regulating the expression of *CHS2*.

## 5. Conclusions

The effects of different salt concentrations and treatment times on the morphology and physiological indices of *R. soongorica* were studied. It was found that low NaCl treatment (100, 200 mM) promoted the growth of *R. soongorica*, while high NaCl treatment (400, 500 mM) inhibited its growth. At the early stage of treatment (24 h), the soluble sugar content in the leaves of *R. soongorica* decreased with the increase in NaCl concentration, which provided an energy source for the smooth progress of various metabolisms. After 72 h of treatment, it increased with the increase in NaCl treatment concentration and then remained at a higher level as an osmotic regulator to maintain osmotic balance in vivo. When treated with 200 mM NaCl for 72 h, *R. soongorica* seedlings showed the strongest salt tolerance. Furthermore, the molecular mechanism of *R. soongorica* leaves was investigated under normal culture (0 mM NaCl) and salt stress treatment (200, 500 mM NaCl) for 72 h. Through transcriptome and proteome association analysis, 40 differential proteins with the same expression trend of differential genes were identified, among which 25 were up-regulated and 15 were down-regulated, and finally, *SYP71*, *CS*, *PCC13-62*, *PASN*, *ZIFL1*, *CHS2*, and other genes were found to be potential target genes for salt tolerance of *R. soongorica*. This study laid a theoretical foundation for further understanding the molecular mechanism of *R. soongorica* in response to salt stress.

## Figures and Tables

**Table 1:** Effects of NaCl stress on the plant height and root length of *R. soongorica* seedlings.

Growth Index	NaCl Concentration (mM·L^−1^)	Treatment Time/h			
		24	72	144	216
Plant height (cm)	0	9.42 ± 0.231 a	10.33 ± 0.207 bc	10.86 ± 0.210 c	12.87 ± 0.378 c
	100	9.77 ± 0.061 a	10.79 ± 0.096 b	11.12 ± 0.253 bc	13.45 ± 0.258 b
	200	9.97 ± 0.558 a	11.37 ± 0.252 a	11.78 ± 0.491 a	14.40 ± 0.150 a
	300	9.70 ± 0.387 a	10.07 ± 0.113 c	11.50 ± 0.452 ab	12.46 ± 0.314 c
	400	9.55 ± 0.444 a	10.12 ± 0.150 c	10.73 ± 0.111 c	11.23 ± 0.469 d
	500	9.35 ± 0.229 a	9.54 ± 0.611 d	10.10 ± 0.155 d	10.59 ± 0.213 e
Root length (cm)	0	8.60 ± 0.200 bc	9.53 ± 0.368 ab	10.42 ± 0.187 a	11.27 ± 0.436 a
	100	8.85 ± 0.141 ab	9.82 ± 0.104 a	10.17 ± 0.262 ab	10.98 ± 0.366 ab
	200	9.20 ± 0.586 a	9.92 ± 0.295 a	10.06 ± 0.213 abc	10.53 ± 0.530 bc
	300	9.40 ± 0.305 a	9.65 ± 0.202 ab	9.82 ± 0.369 bc	9.97 ± 0.337 cd
	400	8.42 ± 0.092 bc	9.2 ± 0.162 bc	9.51 ± 0.413 cd	9.74 ± 0.114 d
	500	8.15 ± 0.218 c	8.78 ± 0.144 c	9.04 ± 0.291 d	9.36 ± 0.240 d

Notes: Data are presented as average ± SE (n = 3). Different lowercase letters denote significant differences at the 0.05 probability level according to the Duncan test.

**Table 2:** DEPs and DEGs with identical expression patterns identified at the mRNA and protein levels in the 0/500 comparison group.

Accession Number	Gene Name	Gene Log_2_ Value	Up/ Down	Protein Fold Change	Up/ Down	Protein Description
D3THI6	UGT71A15	−1.75	down	0.51	down	UDP-glycosyltransferase 71A15
Q9ZSA7	DLO2	−1.93	down	0.53	down	Protein DMR6-LIKE OXYGENASE 2
O24370	LOX2.1	−2.15	down	0.53	down	Linoleate 13S-lipoxygenase 2-1, chloroplastic
Q7X999	RCA2	−1.58	down	0.57	down	Ribulose bisphosphate carboxylase/oxygenase activase 2, chloroplastic
Q8L5A7	SOT15	−2.36	down	0.58	down	Cytosolic sulfotransferase 15
O49675	CCD4	−3.34	down	0.58	down	Probable carotenoid cleavage dioxygenase 4, chloroplastic
P49107	PSAN	−1.86	down	0.59	down	Photosystem I reaction center subunit N, chloroplastic
P27522	CAB8	−1.17	down	0.59	down	Chlorophyll a-b binding protein 8, chloroplastic
P20152	Vim	−2.17	down	0.60	down	Vimentin
K4BW79	EO	−3.53	down	0.61	down	2-methylene-furan-3-one reductase
Q9LR64	PSB27-1	−1.11	down	0.61	down	Photosystem II repair protein PSB27-H1, chloroplastic
Q9SQT8	EMB3004	−2.39	down	0.64	down	Bifunctional 3-dehydroquinate dehydratase/shikimate dehydrogenase, chloroplastic
Q9SSK9	MLP28	−4.00	down	0.65	down	MLP-like protein 28
Q9ZQI8	LTPG12	−2.27	down	0.66	down	Non-specific lipid-transfer protein-like protein At2g13820
P11432	ELIP	2.88	up	1.51	up	PEA early light-induced protein, chloroplastic
O49432	QRT3	1.51	up	1.52	up	Polygalacturonase QRT3
Q9XJ57	CHS2	1.20	up	1.53	up	Chalcone synthase 2
Q9SF29	SYP71	1.03	up	1.56	up	Syntaxin-71
Q9SR86	At3g08860	2.16	up	1.60	up	Alanine--glyoxylate aminotransferase 2 homolog 3, mitochondrial
Q8L856	CYB561A	1.01	up	1.63	up	Transmembrane ascorbate ferrireductase 1
Q08507	ACO3	1.09	up	1.64	up	1-aminocyclopropane-1-carboxylate oxidase 3
Q6Z1G7	Os08g42410	1.20	up	1.65	up	Pyruvate dehydrogenase E1 component subunit beta-1, mitochondrial
Q94JX5	WLIM1	1.10	up	1.73	up	LIM domain-containing protein WLIM1
Q9SXA6	ENDO1	1.79	up	1.78	up	Endonuclease 1
P53800	FDFT	1.02	up	1.84	up	Squalene synthase
A6QP05	DHRS12	1.28	up	1.90	up	Dehydrogenase/reductase SDR family member 12
Q04980	LTI65	1.61	up	2.12	up	Low-temperature-induced 65 kDa protein
P22242	PCC13-62	3.90	up	2.26	up	Desiccation-related protein PCC13-62
Q8LPS2	ACD6	6.09	up	2.51	up	Protein ACCELERATED CELL DEATH 6
Q94BZ1	ZIFL1	2.80	up	2.79	up	Protein ZINC-INDUCED FACILITATOR-LIKE 1
Q55874	sll0103	10.12	up	3.03	up	Uncharacterized protein sll0103
O80433	CS	1.01	up	3.89	up	Citrate synthase, mitochondrial

**Table 3:** KEGG classification for differential protein with the same trend of mRNA expression change in the 0/500 comparison group as in the 0/500 comparison group.

No	Gene Name	Protein Description	KEGG Pathway	Pathway ID
1	LOX2.1	Linoleate 13S-lipoxygenase 2-1, chloroplastic	Linoleic acid metabolism	ko00591
2	LOX2.1	Linoleate 13S-lipoxygenase 2-1, chloroplastic	alpha-Linolenic acid metabolism	ko00592
3	CCD4	Probable carotenoid cleavage dioxygenase 4, chloroplastic	Carotenoid biosynthesis	ko00906
4	PSAN	Photosystem I reaction center subunit N, chloroplastic	Photosynthesis	ko00195
5	CAB8	Chlorophyll a-b binding protein 8, chloroplastic	Photosynthesis—antenna proteins	ko00196
6	PSB27-1	Photosystem II repair protein PSB27-H1, chloroplastic	Photosynthesis	ko00195
7	EMB3004	Bifunctional 3-dehydroquinate dehydratase/shikimate dehydrogenase, chloroplastic	Phenylalanine, tyrosine, and tryptophan biosynthesis	ko00400
8	CHS2	Chalcone synthase 2	Flavonoid biosynthesis	ko00941
9	CHS2	Chalcone synthase 2	Tropane, piperidine, and pyridine alkaloid biosynthesis	ko00960
10	SYP71	Syntaxin-71	SNARE interactions in vesicular transport	ko04130
11	At3g08860	Alanine--glyoxylate aminotransferase 2 homolog 3, mitochondrial	Alanine, aspartate and glutamate metabolism	ko00250
12	At3g08860	Alanine--glyoxylate aminotransferase 2 homolog 3, mitochondrial	Glycine, serine and threonine metabolism	ko00260
13	At3g08860	Alanine--glyoxylate aminotransferase 2 homolog 3, mitochondrial	Cysteine and methionine metabolism	ko00270
14	At3g08860	Alanine--glyoxylate aminotransferase 2 homolog 3, mitochondrial	Valine, leucine and isoleucine degradation	ko00280
15	ACO3	1-aminocyclopropane-1-carboxylate oxidase 3	Cysteine and methionine metabolism	ko00270
16	Os08g42410	Pyruvate dehydrogenase E1 component subunit beta-1, mitochondrial	Glycolysis / Gluconeogenesis	ko00010
17	Os08g42410	Pyruvate dehydrogenase E1 component subunit beta-1, mitochondrial	Citrate cycle (TCA cycle)	ko00020
18	Os08g42410	Pyruvate dehydrogenase E1 component subunit beta-1, mitochondrial	Pyruvate metabolism	ko00620
19	FDFT	Squalene synthase	Steroid biosynthesis	ko00100
20	CS	Citrate synthase, mitochondrial	Citrate cycle (TCA cycle)	ko00020
21	CS	Citrate synthase, mitochondrial	Glyoxylate and dicarboxylate metabolism	ko00630
22	LOX2.1	Linoleate 13S-lipoxygenase 2-1, chloroplastic	Linoleic acid metabolism	ko00591

**Table 4:** Candidate genes and their role in *R. soongorica* tolerance in response to salt stress.

Gene Name	Gene Up/ Down	Protein Up/ Down	Protein Description	Role
PSAN	down	down	Photosystem I reaction center subunit N, chloroplastic	PSAN is a PSI-related gene, and salt stress significantly reduces the photochemical activity of PSI and decreases photosynthetic efficiency. This results in the down-regulation of the PSAN gene and protein expression.
CHS2	up	up	Chalcone synthase 2	Up-regulates CHS2 gene and protein expression and increases secondary metabolites to reduce salt damage.
SYP71	up	up	Syntaxin-71	Under salt stress, to ensure the smooth closure of ion channels and transporters on the plasma membrane and the separation of harmful ions during vesicular transport. By up-regulating the expression of the SYP71 gene and protein, which is involved in the vesicular transport pathway in leaf cells, the effect of Na^+^ and related ions on the growth of *R. soongorica* was mitigated.
PCC13-62	up	up	Desiccation-related protein PCC13-62	The high osmotic effect of salt stress induces physiological drought in the plant root system, and the effect of salt stress on the growth of *R. soongorica* is mitigated by the up-regulation of the PCC13-62 gene and protein expression.
ZIFL1	up	up	Protein ZINC-INDUCED FACILITATOR-LIKE 1	Enhanced growth hormone distribution and signaling to regulate *R. soongorica*’s response to salt stress by up-regulating the ZIFL1 gene and protein expression.
CS	up	up	Citrate synthase, mitochondrial	CS is a core enzyme of the mitochondrial tricarboxylic acid cycle, which is an organic acid that regulates the tricarboxylic acid cycle and directly controls cellular functions. By up-regulating the expression of the CS gene and protein, the adaptation of plant cells to salt stress was improved with various metabolic processes.

## Data Availability

The dataset generated in this study is available under NCBI SRA accession number PRJNA977833, while the mass spectrometry proteomics data have been deposited on ProteomeXchange under accession number PXD042784. Other data are in the supplementary document.

## Supplementary Materials

The following supporting information can be downloaded at: https://www.mdpi.com/article/10.3390/plants12203542/s1, Table S1: Selected genes and their primers in the A vs. B and A vs. C comparison groups; Figure S1: Expression correlation analysis of differential genes in RNA-Seq and qRT-PCR results; Table S2: Expression correlation analysis of differential genes in RNA-Seq and qRT-PCR results; Table S3: Result of RNA-Seq expression profile sequencing; Table S4: Differentially expressed genes in the transcriptome of *Reaumuria soongorica* leaves; Table S5: Differentially expressed proteins in the proteome of *Reaumuria soongorica* leaves; Table S6: Types of protein and mRNA expression changes.

## References

[1] Mukhopadhyay R., Sarkar B., Jat H.S., Sharma P.C., Bolan N.S. (2021). Soil salinity under climate change: Challenges for sustainable agriculture and food security. J. Environ. Manag..

[2] Bhat M.A., Kumar V., Bhat M.A., Wani I.A., Dar F.L., Farooq I., Bhatti F., Koser R., Rahman S., Jan A.T. (2020). Mechanistic Insights of the Interaction of Plant Growth-Promoting Rhizobacteria (PGPR) With Plant Roots Toward Enhancing Plant Productivity by Alleviating Salinity Stress. Front. Microbiol..

[3] Charles S.P., Kominoski J.S., Troxler T.G., Gaiser E.E., Servais S., Wilson B.J., Davis S.E., Sklar F.H., Coronado-Molina C., Madden C.J. (2019). Experimental Saltwater Intrusion Drives Rapid Soil Elevation and Carbon Loss in Freshwater and Brackish Everglades Marshes. Estuaries Coasts.

[4] Arif Y., Singh P., Siddiqui H., Bajguz A., Hayat S. (2020). Salinity induced physiological and biochemical changes in plants: An omic approach towards salt stress tolerance. Plant Physiol. Biochem..

[5] Farhangi-Abriz S., Torabian S. (2017). Antioxidant enzyme and osmotic adjustment changes in bean seedlings as affected by biochar under salt stress. Ecotoxicol. Environ. Saf..

[6] Keunen E.L.S., Peshev D., Vangronsveld J., Van Den Ende W.I.M., Cuypers A.N.N. (2013). Plant sugars are crucial players in the oxidative challenge during abiotic stress: Extending the traditional concept. Plant Cell Environ..

[7] Zayed M., Elkafafi S., Zedan A., Dawoud S. (2017). Effect of Nano Chitosan on Growth, Physiological and Biochemical Parameters of *Phaseolus vulgaris* under Salt Stress. J. Plant Prod..

[8] Tavakoli N.H., Shirvany A., Assareh M.H., Adnani S.M., Mohebbi Kia M. (2019). Evaluation of salinity tolerance in Euphrates poplar (*Populus euphratica Olive.*) ecotypes using stress tolerance indices. J. Arid Biome.

[9] Zhang J., Wang C.X., Wang Y.C., Zheng L.L. (2021). Cloning and functional validation of *RtVAMP2-2* gene of *Reaumuria trigyna*. Pratacultural Sci..

[10] Bansal K.C., Lenka S.K., Mondal T.K., Tuberosa R. (2014). Genomic resources for breeding crops with enhanced abiotic stress tolerance. Plant Breed..

[11] Wang J.C., Meng Y.X., Li B.C., Ma X.L., Lai Y., Si E.J., Yang K.E., Xu X., Shang X., Wang H. (2014). Physiological and proteomic analyses of salt stress response in the halophyte *Halogeton glomeratus*. Plant Cell Environ..

[12] Aliakbari M., Razi H., Alemzadeh A., Tavakol E. (2020). RNA-seq Transcriptome Profiling of the Halophyte *Salicornia persica* in Response to Salinity. J. Plant Growth Regul..

[13] Chen S., Wu F., Li Y., Qian Y., Pan X., Li F., Wang Y., Wu Z., Fu C., Lin H. (2019). NtMYB4 and NtCHS1 Are Critical Factors in the Regulation of Flavonoid Biosynthesis and Are Involved in Salinity Responsiveness. Front. Plant Sci..

[14] Zhang A.Q., Han D.M., Wang Y., Mu H.F., Zhang T., Yan X.F., Pang Q. (2017). Transcriptomic andproteomic feature of salt stress-regulated network in Jerusalem artichoke (*Helianthus tuberosus* L.) root based on de novo assembly sequencing analysis. Planta.

[15] Liu H.H., Chong P.F., Ma Z.Q., Tan B.B., Ma S. (2023). Effects of Exogenous H_2_S on Nitrogen Metabolism in Leaves and Roots of *Reaumuria Soongorica* Seedlings Under Salt Stress. J. Nucl. Agric. Sci..

[16] Zhang H., Liu X., Yang X., Wu H., Zhu J., Zhang H. (2020). miRNA–mRNA Integrated Analysis Reveals Roles for miRNAs in a Typical Halophyte, *Reaumuria soongorica*, during Seed Germination under Salt Stress. Plants.

[17] Yan S.P., Chong P.F., Zhao M. (2022). Effect of salt stress on the photosynthetic characteristics and endogenous hormones, and: A comprehensive evaluation of salt tolerance in *Reaumuria soongorica* seedlings. Plant Signal. Behav..

[18] Zhao X., Yang X.J., Shi Y., He M.M., Tan H.J., Li X.R. (2013). Ion absorption and distribution of symbiotic *Reaumuria soongorica* and *Salsola passerina* seedlings under NaCl stress. Acta Ecol. Sin..

[19] He M.Z., Zhang K., Tan H.J., Hu R., Su J.Q., Wang J., Huang L., Zhang Y.F., Li X.R. (2015). Nutrient levels within leaves, stems, and roots of the xeric species *Reaumuria soongorica* in relation to geographical, climatic, and soil conditions. Ecol. Evol..

[20] Tan H.J., Li X.R., Liu Y.B., Zhao X. (2013). Study on the Antioxidative Ability and Salt Tolerance of *Reaumuria soongorica* Callus under Salt Stress. J. Desert Res..

[21] Liu H.H., Chong P.F., Liu Z.H., Bao X.G., Tan B.B. (2023). Exogenous hydrogen sulfide improves salt stress tolerance of *Reaumuria soongorica* seedlings by regulating active oxygen metabolism. PeerJ.

[22] Zhang J., Cheng K., Wang Y.C. (2023). Analysis of the calcium-dependent protein kinase *RtCDPK16* response to abiotic stress in *Reaumuria trigyna*. Acta Prataculturae Sin..

[23] Tan C., Zhang L., Duan X., Chai X., Huang R., Kang Y., Yang X. (2022). Effects of exogenous sucrose and selenium on plant growth, quality, and sugar metabolism of pea sprouts. J. Sci. Food Agric..

[24] Roshdy A.E.-D., Alebidi A., Almutairi K., Al-Obeed R., Elsabagh A. (2021). The Effect of Salicylic Acid on the Performances of Salt Stressed Strawberry Plants, Enzymes Activity, and Salt Tolerance Index. Agronomy.

[25] Anders S., Pyl P.T., Huber W. (2015). HTSeq—A Python framework to work with high-throughput sequencing data. Bioinformatics.

[26] Chen J., Han G.Q., Shan C., Zhang H.L., Li J.K., Liu H.Y., Zhang Y.X. (2015). Proteomic methods for removing high-abundance proteins in alfalfa leaf. Acta Prataculturae Sin..

[27] Jiang J., Ren X., Li L., Hou R., Sun W., Jiao C., Yang N., Dong Y. (2020). H_2_S Regulation of Metabolism in Cucumber in Response to Salt-Stress Through Transcriptome and Proteome Analysis. Front. Plant Sci..

[28] Zhang M., Chen Z., Yuan F., Wang B., Chen M. (2022). Integrative transcriptome and proteome analyses provide deep insights into the molecular mechanism of salt tolerance in *Limonium bicolor*. Plant Mol. Biol..

[29] Bannari A., Al-Ali Z.M. (2020). Assessing Climate Change Impact on Soil Salinity Dynamics between 1987–2017 in Arid Landscape Using Landsat TM, ETM+ and OLI Data. Remote Sens..

[30] Qi Q., Ma S.R., Xu W.D. (2020). Progress in the study of the effects of salt stress on plant growth and physiological mechanisms of salt tolerance. Mol. Plant Breed..

[31] Stavridou E., Hastings A., Webster R.J., Robson P.R.H. (2017). The impact of soil salinity on the yield, composition and physiology of the bioenergy grass *Miscanthus x giganteus*. GCB Bioenergy.

[32] Sarath N.G., Sruthi P., Shackira A.M., Puthur J.T. (2021). Halophytes as effective tool for phytodesalination and land reclamation. Front. Plant-Soil Interact..

[33] Rahman M.M., Mostofa M.G., Rahman M.A., Miah M.G., Saha S.R., Karim M.A., Keya S.S., Akter M., Islam M., Tran L.-S.P. (2018). Insight into salt tolerance mechanisms of the halophyte *Achras sapota*: An important fruit tree for agriculture in coastal areas. Protoplasma.

[34] Yang S., Zhang H.X., Liu T. (2021). Effect of Salt Stress on Osmotic Adjustment Substances in Plants. For. Res..

[35] Wu H.X., Jia H.M., Ma X.W., Wang S.B., Yao Q.S., Xu W.T., Zhou Y.G., Gao Z.S., Zhan R.L. (2014). Transcriptome and proteomic analysis of mango (*Mangifera indica* Linn) fruits. J. Proteom..

[36] Ding H.D., Mo S.R., Qian Y., Yuan G.B., Wu X.X., Ge C.L. (2020). Integrated proteome and transcriptome analyses revealed key factors involved in tomato (*Solanum lycopersicum*) under high temperature stress. Food Energy Secur..

[37] Yoon T.Y., Munson M. (2018). SNARE complex assembly and disassembly. Curr. Biol..

[38] Baral A., Shruthi K.S., Mathew M.K. (2015). Vesicular trafficking and salinity responses in plants. IUBMB Life.

[39] Salinas-Cornejo J., Madrid-Espinoza J., Ruiz-Lara S. (2019). Identification and transcriptional analysis of SNARE vesicle fusion regulators in tomato (*Solanum lycopersicum*) during plant development and a comparative analysis of the response to salt stress with wild relatives. J. Plant Physiol..

[40] Suwastika I.N., Uemura T., Shiina T., Sato M.H., Takeyasu K. (2008). SYP71, a plant-specific Qc-SNARE protein, reveals dual localization to the plasma membrane and the endoplasmic reticulum in *Arabidopsis*. Cell Struct. Funct..

[41] Bao Y.M., Sun S.J., Li M., Li L., Cao W.L., Luo J., Tang H.J., Huang J., Wang Z.F., Wang J.F. (2012). Overexpression of the Qc-SNARE gene *OsSYP71* enhances tolerance to oxidative stress and resistance to rice blast in rice (*Oryza sativa* L.). Gene.

[42] Kang W., Harada Y., Yamatoya K., Kawano N., Kanai S., Miyamoto Y., Nakamura A., Miyado M., Hayashi Y., Kuroki Y. (2020). Extra-mitochondrial citrate synthase initiates calcium oscillation and suppresses age-dependent sperm dysfunction. Lab. Investig..

[43] Zhang Z., Mao C.Y., Shi Z., Kou X.H. (2017). The Amino Acid Metabolic and Carbohydrate Metabolic Pathway Play Important Roles during Salt-Stress Response in Tomato. Front. Plant Sci..

[44] Tahjib-Ul-Arif M., Zahan M.I., Karim M.M., Imran S., Hunter C.T., Islam M.S., Mia M.A., Hannan M.A., Rhaman M.S., Hossain M.A. (2021). Citric Acid-Mediated Abiotic Stress Tolerance in Plants. Int. J. Mol. Sci..

[45] Weng Q.Y., Zhao Y.M., Zhao Y.N., Song X., Yuan J.C., Liu Y.H. (2021). Identification of Salt Stress-Responsive Proteins in Maize (*Zea may*) Seedlings Using iTRAQ-Based Proteomic Technique. Iran. J. Biotechnol..

[46] Panda A., Rangani J., Parida A.K. (2020). Comprehensive proteomic analysis revealing multifaceted regulatory network of the xero-halophyte *Haloxylon salicornicum* involved in salt tolerance. J. Biotechnol..

[47] Wang Y.N., Li K.X., Li X. (2009). Auxin redistribution modulates plastic development of root system architecture under salt stress in *Arabidopsis thaliana*. J. Plant Physiol..

[48] Haydon M.J., Cobbett C.S. (2007). A Novel Major Facilitator Superfamily Protein at the Tonoplast Influences Zinc Tolerance and Accumulation in *Arabidopsis*. Plant Physiol..

[49] Remy E., Cabrito T.R., Baster P., Batista R.A., Teixeira M.C., Friml J., Sá-Correia I., Duque P. (2013). A Major Facilitator Superfamily Transporter Plays a Dual Role in Polar Auxin Transport and Drought Stress Tolerance in *Arabidopsis*. Plant Cell.

[50] Remy E., Baster P., Friml J., Duque P. (2014). ZIFL1.1 transporter modulates polar auxin transport by stabilizing membrane abundance of multiple PINs in *Arabidopsis* root tip. Plant Signal. Behav..

[51] Zha H.G., Liu T., Zhou J.J., Sun H. (2013). MS-desi, a desiccation-related protein in the floral nectar of the evergreen velvet bean (*Mucuna sempervirens* Hemsl): Molecular identification and characterization. Planta.

[52] Li W., Zhao F.a., Fang W., Xie D., Hou J., Yang X., Zhao Y., Tang Z., Nie L., Lv S. (2015). Identification of early salt stress responsive proteins in seedling roots of upland cotton (*Gossypium hirsutum* L.) employing iTRAQ-based proteomic technique. Front. Plant Sci..

[53] Giarola V., Jung N.U., Singh A., Satpathy P., Bartels D. (2018). Analysis of *pcC13-62* promoters predicts a link between cis-element variations and desiccation tolerance in Linderniaceae. J. Exp. Bot..

[54] Zhang H.H., Shi G.L., Shao J.Y., Li X., Li M.B., Meng L., Nan X., Guang-Yu S. (2019). Photochemistry and proteomics of mulberry (*Morus alba* L.) seedlings under NaCl and NaHCO_3_ stress. Ecotoxicol. Environ. Saf..

[55] Yue J., Shi D., Zhang L., Zhang Z., Fu Z., Ren Q., Zhang J. (2020). The photo-inhibition of camphor leaves (*Cinnamomum camphora* L.) by NaCl stress based on physiological, chloroplast structure and comparative proteomic analysis. PeerJ.

[56] Pandith S.A., Ramazan S., Khan M.I., Reshi Z.A., Shah M.A. (2019). Chalcone synthases (CHSs): The symbolic type III polyketide synthases. Planta.

[57] Tian L., Kong W.F., Pan Q.H., Zhan J.C., Wen P.F., Chen J.Y., Wan S.B., Huang W.D. (2006). Expression of the chalcone synthase gene from grape and preparation of an anti-CHS antibody. Protein Expr. Purif..

[58] Marriboina S., Sekhar K.M., Subramanyam R., Reddy A.R. (2022). Physiological, Biochemical, and Root Proteome Networks Revealed New Insights into Salt Tolerance Mechanisms in *Pongamia pinnata* (L.) Pierre. Front. Plant Sci..

