# Supplementary material for: Transcriptome and Proteome Association Analysis to Screen Candidate Genes Related to Salt Tolerance in Reaumuria soongorica Leaves under Salt Stress
Source: Plants (Basel). 2023 Oct 12;12(20):3542. doi: 10.3390/plants12203542 (PMC10609793; doi:10.3390/plants12203542)
Supplement: Supplementary file 1 [file plants-12-03542-s001.zip › Table S1 Selected genes and their primers in the A vs B and A vs C comparison groups.pdf]

Table S1 Selected genes and their primers in the A vs B and A vs C comparison groups

Gene ID	Forward primer (5'-3')	Reverse primer (5'-3')
DN1935_c1_g1	TGCTGGTGACACTCATCTCGGGGGA	AAGTGCCCTGGGGTTGCCACTGAT
DN550_c1_g3	TGCCGGGGGTATACCAGAGAGCTCA	ACTGCTGGAGGAGTCATGACCGT
DN4132_c0_g1	TGCATGCGAAGTACCGTCGGCA	ACTAGGGCAACTGTCGCTACCGA
DN18860_c0_g1	ACGGAGAGTTGCGAAAGGAGACGGTGC	TTCTATTGCAGCCACATGCCGCTCCGC
DN1826_c0_g1	GCTGCAACTCTTTGTGTGTGTCGGCA	TCCCCGGCTTCCTGATTTCCGA
DN8009_c0_g1	ATCCACAACCGCCGCCACAT	TGGACGGCCTGAAGAAGAGCCT
DN10354_c0_g1	TACGTAGACTGCAACGACGAGGGCGCA	ACGCCAATAGCTAGTCCGCCGCAGT
DN1062_c0_g1	ACACCGCATGTTTGGTTGCCCGGGA	AATGCCCGAACCAGACGCAACCCCT
DN5051_c1_g1	TGCTCTGCTGCTGCTGAAGGGA	TCGGGCCTGCTTCTGTTCTGCT
DN3844_c0_g1	AACCCACCAACCCAGACAGCCT	TCCCAGCACCCCGCCCTTGGAATTT
DN4083_c0_g1	AGTGCGGCTGGTTGCATGGACA	TCCACCCCGTCAATCAGTCCGA
DN16513_c0_g1	ACGCAGTTCAAGAGCAACCGCA	TCAGCACAGGAGAAGGACGCCCCAA
DN1935_c1_g1	TGCTGGTGACACTCATCTCGGGGGA	AAGTGCCCTGGGGTTGCCACTGAT
DN831_c0_g1	TGGATCTCGGGTTGCCACCTTGGGA	AGCGCCACACCAACAATGCCGGGTA
DN3504_c0_g1	AGAGGTTGTATGCTGGCCGGGA	AGTTCTCCGCTGTCGAACTCCGGCT
DN6988_c0_g1	AGAATTGGTCTCGGAGCTGGCGCCT	ACTTTTCCTCGCAGGCGCTCGCTT
DN12763_c0_g1	CAGCAAGGTGCCCGCTTCGCCAAAT	AACGAGCAAGACCCCAGGCTGCTT
DN7885_c1_g1	TGGTGGCAAAAGCCCAAGCGT	TGATCCCGAGGCATTTGCTGAGCT
DN5144_c0_g1	AAGTGGACACGGCAGTTCTGGTGGC	TTACCGGTGCCATCGCTTGCGACCA
DN2339_c0_g1	ATTGCGATGGTACGGGCGGGCTCAA	AGGGACTGCGGGGATACAGGAGAAGCT
