# Supplementary material for: Transcriptome and Proteome Association Analysis to Screen Candidate Genes Related to Salt Tolerance in Reaumuria soongorica Leaves under Salt Stress
Source: Plants (Basel). 2023 Oct 12;12(20):3542. doi: 10.3390/plants12203542 (PMC10609793; doi:10.3390/plants12203542)
Supplement: Supplementary file 1 [file plants-12-03542-s001.zip › Table S2 Expression correlation analysis of differential genes in RNA-Seq and qRT-PCR results.pdf]

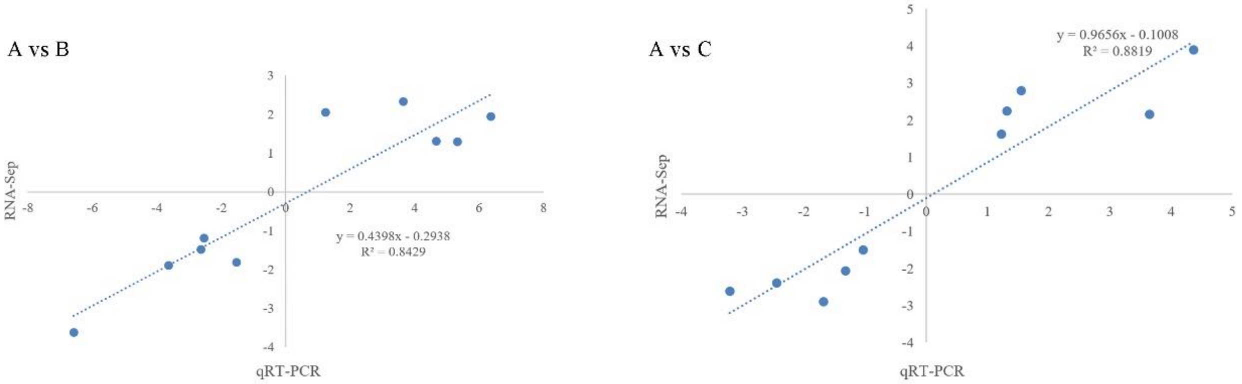

Figure S1 Expression correlation analysis of differential genes in RNA-Seq and qRT-PCR results

Table S2 Differential gene expression in RNA-Sep and qRT-PCR

**A vs B**			**A vs C**		
Gene-ID	qRT-PCR	RNA-Sep	Gene-ID	qRT-PCR	RNA-Sep
DN1935_c1_g1	6.36	1.949685238	DN4083_c0_g1	4.36	3.903829518
DN1826_c0_g1	5.33	1.29788382	DN1935_c1_g1	3.64	2.166039053
DN4132_c0_g1	4.66	1.309714595	DN16513_c0_g1	1.55	2.799154244
DN18860_c0_g1	3.65	2.335323778	DN831_c0_g1	1.31	2.246444044
DN550_c1_g3	1.23	2.053450844	DN3504_c0_g1	1.22	1.624168258
DN3844_c0_g1	-2.54	-1.182893932	DN2339_c0_g1	-1.03	-1.491428659
DN1062_c0_g1	-1.53	-1.813380528	DN6988_c0_g1	-1.32	-2.057890026
DN10354_c0_g1	-6.58	-3.624512899	DN7885_c1_g1	-1.68	-2.894573157
DN8009_c0_g1	-2.64	-1.48050298	DN5144_c0_g1	-2.45	-2.390644772
DN5051_c1_g1	-3.65	-1.896520183	DN12763_c0_g1	-3.21	-2.604786217
