# Supplementary material for: Transcriptome and Proteome Association Analysis to Screen Candidate Genes Related to Salt Tolerance in Reaumuria soongorica Leaves under Salt Stress
Source: Plants (Basel). 2023 Oct 12;12(20):3542. doi: 10.3390/plants12203542 (PMC10609793; doi:10.3390/plants12203542)
Supplement: Supplementary file 1 [file plants-12-03542-s001.zip › Table S3 Result of RNA-seq expression profile sequencing.pdf]

Table S3 Result of RNA-seq expression profile sequencing

NaCl concentration（$mM \cdot L^{-1}$）	Sample	Total Reads	Total Base Pairs/bp	Clean Reads	Clean Base/bp	Q30 (%)
0	A1	56008922	8401338300	50690698	7603604700	94.51
	A2	48145618	7221842700	43921906	6588285900	94.64
	A3	46287448	6943117200	41773372	6266005800	94.71
200	B1	47016140	7052421000	41825054	6273758100	94.73
	B2	46429918	6964487700	42082904	6312435600	94.39
	B3	44631734	6694760100	40619710	6092956500	94.41
500	C1	46516972	6977545800	42107346	6316101900	94.68
	C2	46248756	6937313400	42207874	6331181100	93.76
	C3	47819874	7172981100	43560466	6534069900	94.55

Note：

Sample：the name of the sample.

Total Reads：total number of Reads.

Total Base Pairs (bp)：total number of bases.

Clean Reads：the number of high-quality sequence reads.

Clean Data (bp)：number of bases of high-quality sequences.

Q30 (%)：The percentage of bases whose base recognition accuracy is above 99.9%.
